# Forest Age and Plant Species Composition Determine the Soil Fungal Community Composition in a Chinese Subtropical Forest

**DOI:** 10.1371/journal.pone.0066829

**Published:** 2013-06-27

**Authors:** Yu Ting Wu, Tesfaye Wubet, Stefan Trogisch, Sabine Both, Thomas Scholten, Helge Bruelheide, François Buscot

**Affiliations:** 1 UFZ-Helmholtz Centre for Environmental Research, Department of Soil Ecology, Halle (Saale), Germany; 2 Chair of Soil Ecology, Institute of Biology, University of Leipzig, Leipzig, Germany; 3 German Centre for Integrative Biodiversity Research (iDiv), Leipzig, Germany; 4 Faculty of Biology, Department of Geobotany, University of Freiburg, Freiburg, Germany; 5 Department of Biology and Geobotany, Martin Luther University Halle Wittenberg, Halle (Saale), Germany; 6 Chair of Physical Geography and Soil Science, University of Tübingen, Tübingen, Germany; Wageningen University, The Netherlands

## Abstract

Fungal diversity and community composition are mainly related to soil and vegetation factors. However, the relative contribution of the different drivers remains largely unexplored, especially in subtropical forest ecosystems. We studied the fungal diversity and community composition of soils sampled from 12 comparative study plots representing three forest age classes (Young: 10–40 yrs; Medium: 40–80 yrs; Old: ≥80 yrs) in Gutianshan National Nature Reserve in South-eastern China. Soil fungal communities were assessed employing ITS rDNA pyrotag sequencing. Members of Basidiomycota and Ascomycota dominated the fungal community, with 22 putative ectomycorrhizal fungal families, where *Russulaceae* and *Thelephoraceae* were the most abundant taxa. Analysis of similarity showed that the fungal community composition significantly differed among the three forest age classes. Forest age class, elevation of the study plots, and soil organic carbon (SOC) were the most important factors shaping the fungal community composition. We found a significant correlation between plant and fungal communities at different taxonomic and functional group levels, including a strong relationship between ectomycorrhizal fungal and non-ectomycorrhizal plant communities. Our results suggest that in subtropical forests, plant species community composition is the main driver of the soil fungal diversity and community composition.

## Introduction

Fungi are a highly diverse component of soil microbial communities. They play essential roles in many aspects of ecosystem development, functioning and stability [1]. As pathogens and symbionts, soil fungi influence the species composition and dynamics of plant communities [2], [3]; as saprotrophs, they are an essential component of the soil food web influencing nutrient cycling and carbon sequestration [4]. About 100,000 fungal species have been described, but it has been estimated that there may be from 1.5 to 5.1 million extant fungal species [5]. Most investigations on fungal diversity and distribution have been accomplished in temperate, tropical and boreal regions (e.g. [6]–[8]). Consequently, there is a limited knowledge in the subtropics, although subtropical forest ecosystems are among the most prominent plant diversity hotspots in the holarctic realm [9].

It has been suggested that high plant diversity supports high fungal diversity [6], [10], [11], implying that subtropical ecosystems might harbor a high fungal diversity [12]. Recent studies on the fungal diversity in Chinese subtropical forests focused on fungal biomass [13], ECM sporocarp and mycorrhizal root analyses [14]–[16], and on the cultivable active microbial community [17], but there is still a gap of knowledge on the soil fungal diversity and the factors influencing the community composition.

In forest ecosystems, fungal diversity and community composition have been reported to be closely linked to numerous biotic and abiotic factors, such as stand age [17], [18], elevation [19], [20], plant diversity [6], [21], plant productivity [2], [22], and soil environment [23], [24]. The impact of these factors on the soil fungal community has rarely been studied in combination, particularly in the very species-rich subtropical natural forests. In forest communities dominated by only one or a few tree species, such as temperate or boreal forests, it is expected that the soil fungal community is strongly dependent on the identity of a particular tree species. For example, it has been shown that different combinations of plant species have a paramount influence on soil fungal community composition [25]. Consequently, species identity effects of a particular tree species cannot be separated from diversity effects (compare [26]). In tropical forests with 6 to 9 different species per 10 sampled individuals [27] and in subtropical forests with 4.4 to 8.3 different species per 10 sampled individuals, as is the case in our study area (Helge Bruelheide, personal communication), plant species identity effects are expected to be an important factor influencing the fungal community.

In general, a high diversity in plant species is expected to result in high diversity levels at other trophic levels, as diverse plant communities offer a larger amount and larger heterogeneity of resources to consumers, parasites and symbionts [28], [29]. In contrast to simply focusing on richness relationships, comparisons of communities at different trophic levels have the advantage that the data also accounts for structural factors and idiosyncratic links between species. The latter seems to be particularly important for the analysis of specificity in plant species - microbial community relationships [30], [31], and suggests concordance in the composition of tree species and fungal communities.

We assessed the diversity and community composition of soil fungi using a natural species richness gradient along a chronosequence representing three forest age classes in a Chinese subtropical forest located in the Gutianshan National Nature Reserve (Zhejiang, China). The study plots were established in the framework of the Biodiversity and Ecosystem Functioning project (BEF-China) [32]. As soil parent material and other topographic variables along this chronosequence did only vary to a minor degree, we expected that most of the variation in the fungal community composition should be caused by the biotic components of the ecosystem. To obtain sufficient depth in our analyses, we employed massively parallel and targeted ITS rDNA pyrotag sequencing, an approach currently used in a number of studies investigating soil bacterial [33], [34] and fungal communities [35], [36]. The objectives of this study were to characterize fungal diversity in this subtropical forest ecosystem, to assess the fungal community composition among the forest age classes, and analyze the contribution of the soil environment, plant species richness and composition on the fungal community. In particular, we hypothesized that 1) fungal community composition differs among the forest age classes, 2) plant cover and variables related to plant biomass explain most of the variation in the soil fungal community composition, and 3) that there is a positive relationship between plant species and soil fungal community composition.

## Materials and Methods

### Ethics Statement

All necessary permits for the described field studies were issued by the Administration Bureau of the Gutianshan National Nature Reserve, Zhejiang, China.

### Study Site and Soil Sampling

The study was conducted at the Gutianshan National Nature Reserve (NNR) in the Zhejiang Province in south-eastern China (29°8'18" –29°17'29" N, 118°2'14" –118°11'12" E). The NNR is approximately 81 km^2^ in size and is located in a mountainous region with a typical subtropical climate. The mean annual temperature at the NNR is 15.3°C with a maximum of 38.1°C in July and a minimum of −6.8°C in January [37]. The annual mean precipitation is 1964 mm (calculated based on data from 1958 to 1986), occurring mostly between March and September [38]. Approximately 57% of the reserve is natural forest [38]. The NNR vegetation type is representative of typical subtropical forest ecosystems consisting of mixed evergreen broad-leaved species. A total of 111 woody species, including 24 ectomycorrhizal (ECM) and 87 non ECM tree species, from 41 families have been recorded at the study plots, with *Castanopsis eyrei* (*Fagaceae*), *Daphniphyllum oldhamii* (*Daphniphyllaceae*), and *Schima superba* (*Theaceae*) as the dominant woody species [32].

The study was carried out in 12 Comparative Study Plots (CSPs), which were randomly selected and stratified by successional age [32] (Fig. S1). Each CSP had a size of 30 m×30 m, divided into 9 subplots with 10 m×10 m size representing three age classes since natural regeneration (Young: 10–40 yrs; Medium: 40–80 yrs; Old: ≥ 80 yrs, with four replicate plots each). Abundance of all tree (>1 m in height) species was assessed in the entire plot, while herb species abundance (<1 m in height) was only surveyed in the central subplot. Forest floor litter biomass was determined with a PVC ring (19 cm in diameter) driven into the undisturbed litter layer in spring 2009. In each CSP, 4 litter cores were taken at randomly selected locations (total area of the 4 cores = 0.11 m^2^) and pooled for dry weight measurement. Soil sampling was carried out between March and April 2009, where within each CSP soil samples were collected to 10 cm depth in each of the 9 subplots using an auger with a 10-cm diameter. The 9 subsamples were then bulked into one composite sample per study plot. Homogenized samples were immediately hand-sieved (≤2 mm) in the field to remove stones, roots, macrofauna, and litter materials. Subsamples were frozen in liquid nitrogen, transported in an ice box and were stored at −20°C until molecular analysis. The analysis methods of soil characteristics and the vegetation data (presented in Table 1) are described in detail in [13].

**Table 1:** Elevation of study sites, plant parameters and soil characteristics of the comparative study plots (CSPs) and their relationships with forest age class based on Pearson correlation analysis.

Forest age	CSP16	CSP17	CSP25	CSP26	CSP01	CSP05	CSP08	CSP09	CSP02	CSP04	CSP12	CSP13	Pearson R^2^
	Young	Young	Young	Young	Medium	Medium	Medium	Medium	Old	Old	Old	Old	
Elevation (m)	309	310	345	251	522	507	413	348	390	542	620	402	**0.705** **
**Plant parameters**													
***Plant cover***													
Herb layer cover (%)	55	10	60	10	5	5	45	50	10	10	4	15	−0.427
Tree layer cover (%)	64	60	33	48	30	25	33	30	23	33	30	25	**−0.754** **
Deadwood cover (%)	1	3	2	4	25	15	10	4	10	10	90	10	**0.712** **
Opensoil cover (%)	1	15	0	10	10	3	2	8	20	1	3	2	0.04
Rock cover (%)	0	0	0	1	0	1	0	0	3	10	0	0	0.432
Litter layer (thickness, cm)	2.79	2.46	2.92	2.25	3.04	2.46	4.21	3.50	3.71	3.08	2.96	2.79	0.429
***Plant richness***													
Herb species richness	17	12	8	14	11	11	12	17	17	8	6	12	−0.271
Tree species richness	37	39	27	44	44	25	53	55	69	44	29	32	0.173
***Plant biomass***													
Herbaceous biomass (g)	26.3	5.21	64.2	5.49	9.08	0.23	25.9	38.0	9.07	5.15	0.00	0.64	−0.512
Woody plant biomass (<1 m) (g)	16.7	12.7	10.4	7.82	9.16	2.09	2.56	0.21	0.23	4.12	0.19	8.07	**−0.631** *
Litter biomass (dry weight, g per 0.11 m^2^)	69.4	101.0	85.4	87.4	122.6	80.0	92.9	60.9	78.3	97.2	63.5	89.9	−0.099
**Soil characteristics**													
Sand (%)	52.7	47.3	54.2	55.6	48.1	53.8	48.1	42.2	35.3	31.4	45.9	53.0	**−0.611** *
Clay (%)	20.4	23.9	18.8	19.9	20.4	18.0	20.5	22.3	24.8	23.7	16.4	19.3	0.015
Soil organic carbon (%)	4.05	4.51	4.07	4.11	5.70	4.03	7.11	7.22	7.43	8.54	6.15	3.55	0.535
C/N ratio	19.1	17.9	21.6	16.1	18.1	19.5	20.1	18.0	15.9	15.8	18.3	18.7	−0.364
pH_KCl_	3.82	3.84	3.73	4.01	3.75	3.87	3.78	3.61	3.75	3.72	3.95	3.94	−0.038

Significant values (Bonferroni corrected *P*<0.05) are shown in bold.

* P<0.05,

** P<0.01.

### DNA Extraction, Amplicon Library Generation and Pyrosequencing

Soil microbial genomic DNA was extracted from approximately 1 g (wet weight) of soil using a MoBio PowerSoil DNA Isolation Kit (MoBio Laboratories Inc. Carsbad, CA, USA) following the manufacturer’s instructions. Fungal ITS rDNA amplicon libraries were produced using fusion primers [39] designed with pyrosequencing primer B, a barcode and the fungal specific primer ITS1F [40] as a forward primer and pyrosequencing primer A and the universal eukaryotic primer ITS4 [41] as a reverse primer. We used a set of 10 bp MID-barcodes provided by Roche (Roche Applied Science). ITS rDNA amplicon libraries were produced from a pool of two dilution levels (10× and 100×) from each soil DNA extract. The PCRs were performed in three replicate reactions per sample and per dilution to account for potentially heterogeneous amplification.

PCR reaction was carried out in a total volume of 50 µl containing 1 µl diluted DNA template, 1 µl 25 pmol of each of the two custom fusion primers, 25 µl Go Tag^®^ Green Master mix (Promega), and nuclease free water (Promega). We used a touchdown PCR program with a denaturation at 95°C for 5 min followed by 10 cycles of denaturation at 94°C for 30 sec, annealing at 60–50°C for 45 sec (−1°C per cycle), and extension at 72°C for 2 min; and then 30 cycles at 94°C for 30 sec, 50°C for 45 sec and 72°C for 2 min, then finalized by an extension step at 72°C for 10 min. PCR products were analyzed using 1.5% agarose gel and equimolar volumes of the amplified products of the expected size (ca. 600 bp) from the three positive replicate amplicons per sample were homogenized. The pooled products were gel purified using a Qiagen Gel Extraction Kit (Qiagen, Hilden, Germany). The amount of DNA in the purified amplicons was quantified using a fluorescence spectrophotometer (Cary Eclipse, Agilant Technologies, Waldbronn, Germany). An equimolar mix of the 12 amplicon libraries was subjected to unidirectional pyrosequencing from the ITS1F end of the amplicons using a 454 titanium amplicon sequencing kit and a Genome Sequencer FLX 454 System (454 Life Sciences/Roche Applied Biosystems, Mannheim, Germany) at the Department of Soil Ecology, Helmholtz Centre for Environmental Research (UFZ, Halle/Germany).

### Bioinformatics Analysis

Multiple levels of sequence processing and quality filtering were performed as described in [42]. Briefly the 454 fungal ITS sequences were initially extracted based on 100% barcode similarity. Simultaneously, sequence reads with an average quality score of less than 25, read length of <200 bp, ambiguous bases and homo-polymers of >8 bases were removed, barcodes and primers were trimmed using the split libraries script available in the Quantitative Insights In Microbial Ecology pipeline (QIIME) [43]. Based on preliminary sequence analysis, reads >450 bp were trimmed to a maximum read length of 450 bp using MOTHUR [44]. Sequences were then clustered and assigned to operational taxonomic units (OTU) using the QIIME implementation of CDHIT with a threshold of 97% pairwise identity after a pre-filtering step to remove identical sequences using default parameters. The most abundant representative sequences were selected and assigned to respective taxa according to NCBI taxonomy. The searches were based on blastn against the NCBI nucleotide database for fungi excluding uncultured and environmental sequences using the blast based taxonomic assignment script of the software for **C**leaning and **A**nalyzing **N**ext **G**eneration **S**equences - CANGS [45].

Representative sequences of OTUs of the fungal ITS pyrotags assigned under the fungal kingdom were checked for chimeras using the chimera uchime algorithm using the same dataset as a reference, as implemented in MOTHUR. Subsequently a total of 2398 sequences identified as potentially chimeric and had less than 90% alignment length to sequences in the NCBI fungal reference database, and 1445 reads not assigned to the fungal kingdom were removed from the sequence dataset.

Finally, we found 14,136 sequences that were grouped into 1260 OTUs with variable number of reads per sample (Table S1). Consequently, the number of sequences per sample was normalized to the smallest sample size, 872 reads per sample, using the normalized shared command as implemented in MOTHUR.

### Ectomycorrhizal Fungi Designation

The Ascomycotan and Basidiomycotan fungal OTUs were further identified as putative ectomycorrhizal (ECM) fungi at the genus level based on literature [46]–[50] as described in [50]. All ECM genera which were presented as non mycorrhizal (NM) by Tedersoo et al. [50] in contrast to Rinaldi et al. [48], were treated as NM. Furthermore, we did a manual NCBI blast search for the representative sequences of those genera reported to be composed of both ectomycorrhizal and saprotrophic species. Accordingly only those OTUs with blast similarities of >97% with sequences derived from mycorrhizal roots were maintained in our final putative ECM fungal dataset.

### Statistical Analysis

The fungal OTUs were parsed by sample in order to calculate the abundance of fungal OTUs using the sequence count of each of the non-singleton OTUs as abundance value [42], [51]. Based on a preliminary rank index analysis, we calculated dissimilarities between all pairs of samples using the log (x+1) transformed abundance data and Bray-Curtis dissimilarity coefficient in order to obtain an abundance based dissimilarity matrix. To assess the effect of singletons on the fungal community distribution, we calculated the non-metric multidimensional scaling (NMDS) ordinations with 20 random starts from the datasets with and without singletons. The correlation between the ordinations was tested using the Procrustes correlation analysis using the protest function [52] of the vegan package [53], where the significance of the congruence between any two ordinations was tested by a Monte Carlo procedure with 999 permutations. We found that the fungal community composition was not affected by the presence or absence of singletons (Procrustes correlation coefficient = 0.966, *P*<0.001, suggesting nearly identical ordinations). Similar analysis using the presence or absence dataset also showed that the NMDS ordinations were significantly correlated (Procrustes correlation coefficient = 0.97, *P*<0.001). Thus we performed the subsequent analyses using the dominant fungal community excluding singletons.

We used the function ANOSIM of the vegan package to explore the similarity of fungal community composition among the forest age classes. Alpha and beta diversity based fungal community compositions across the three age classes were compared using the abundance-based pair-wise Bray-Curtis dissimilarity and the Sorenson pair-wise dissimilarity matrix accounting for beta diversity using the betapart package [54]. The relative abundance of ECM fungi at the family level and the distribution of one of the most abundant ECM family at the OTU level across the three forest age classes was visualized by a heatmap using the function heatmap2 of the gplots package [55].

The vegetation and soil characteristics were checked for collinearity with the function varclus in the Hmisc package [56], using Spearman’s rank correlation. The resulting set of non-collinear parameters was maintained for further analyses (Table 1 and Fig. S2). Based on preliminary rank index analysis of these exploratory variables, non-metric multidimensional scaling (NMDS) analysis was performed using the nmds function of the labdsv package [57] based on Bray-Curtis distances to visualize the distribution of the fungal communities. The function envfit of vegan was used to calculate a post-hoc regression of the individual environmental variables on the ordination scores. R^2^ or the goodness-of-fit values and their significances were calculated using 999 random permutations. Pearson correlation coefficients were calculated to elucidate patterns of correlations of environmental variables using the function corr.test of the Psych package [58].

Model of multivariate analysis of variance was constructed using distance based redundancy analysis (dbRDA) using Bray–Curtis distances with the function capscale of vegan to determine the most influential environmental variables on the fungal community composition. Permutational multivariate analysis of variance (Permanova) was used to determine the interaction of the selected variables and assess their influence on the fungal community composition using the adonis function of vegan with the Bray-Curtis distances and 999 permutations.

The concordance of plant and fungal community ordinations was assessed using Procrustes correlation analysis as described above using plant and fungal NMDS ordinations. The contribution of individual soil characteristics to the plant and fungal community congruence was tested using ANOVA of Procrustes residuals. Based on the preliminary normality tests, in all the above analysis both plant species abundances and environmental variables were log (x+1) transformed. Statistical analysis was performed using R version 2.15.2 [59].

## Results

### Pyrosequencing Data Analysis and Taxonomic Assignment

A total of 16,534 fungal ITS pyrotag reads with an average length of 450 bp were obtained from the 12 soil samples collected across the three forest age classes. After multiple levels of sequence processing, quality filtering, and sequence number normalization followed by a 3% dissimilarity clustering we found 1027 fungal OTUs including 457 (44%) singletons from 10,464 fungal ITS reads (Table S1). Taxonomic distribution of the 570 abundant fungal OTUs, excluding the singletons, showed distribution of the fungal community mainly across four fungal phyla with 50.2% belonging to Basidiomycota, 37.3% to Ascomycota, 2.3% to Zygomycota, and 0.4% to Glomeromycota. We were able to assign 73.7% and 68.8% of these OTUs to the family and genus levels, respectively. The remaining 9.8% OTUs were grouped as unidentified fungi indicating the presence of unknown fungal taxa in this particular subtropical forest ecosystem.

Within the two major fungal phyla, the Ascomycota comprised of 213 OTUs, which were mainly members of the subphylum Pezizomycotina (180/213). A total of 35 ascomycetous families were identified among which the *Trichocomaceae*, *Myxotrichaceae*, *Elaphomycetaceae*, *Hypocreaceae*, and *Herpotrichiellaceae* were the most represented. From the total of 286 OTUs identified as members of the phylum Basidiomycota, the majority (282 OTUs) was assigned to the subphylum Agaricomycotina. A total of 36 basidiomycetous families were found, with *Russulaceae*, *Sebacinaceae*, *Cortinariaceae*, *Thelephoraceae*, *Amanitaceae*, *Tricholomataceae*, and *Clavulinaceae* being the most abundant ones.

It is also noteworthy that, within the two dominant fungal phyla, 10% of the 213 ascomycetes and 75% of the 286 basidiomycetes fungal OTUs were identified as potential ectomycorrhizal (ECM) fungi. The ECM fungal OTUs (234 OTUs) represented 22 families (5 belonging to Ascomycota and 17 to Basidiomycota) and 34 genera (5 Ascomycota and 29 Basidiomycota). The relative abundance of ECM fungal communities at the family level showed that *Russulaceae*, *Thelephoraceae*, *Helotiaceae, Amanitaceae*, *Sebacinaceae*, *Entolomataceae*, *Cortinariaceae*, *Elaphomycetaceae and Ceratobasidiaceae* were the most abundant families across the three forest age classes. At the genus level, *Russula*, *Tomentella*, *Amanita*, *Thelephora*, *Entoloma*, *Sebacina*, and *Cortinarius* were the most abundant (Fig. S3 a, b).

### Fungal Community Assemblage across the Three Forest Age Classes

The presence or absence of singletons showed no significant effect on the fungal community ordination at the fungal Kingdom level (see Material and Methods). These ordinations were also consistent when confined to the phylum Ascomycota (Procrustes correlation coefficient = 0.742, *P*<0.01), phylum Basidiomycota (Procrustes correlation coefficient = 0.929, *P*<0.001), and ECM fungi (Procrustes correlation coefficient = 0.985, *P*<0.001) levels tested. Analysis of similarity of the dominant fungal community assemblages revealed significant differences of the fungal community across the three forest age classes (ANOSIM, R = 0.345, *P<*0.01), which was consistent in the phylum Basidiomycota (ANOSIM, R = 0.24, *P<*0.05) and ECM (ANOSIM, R = 0.36, *P<*0.05) fungal communites.

**Figure 1:**
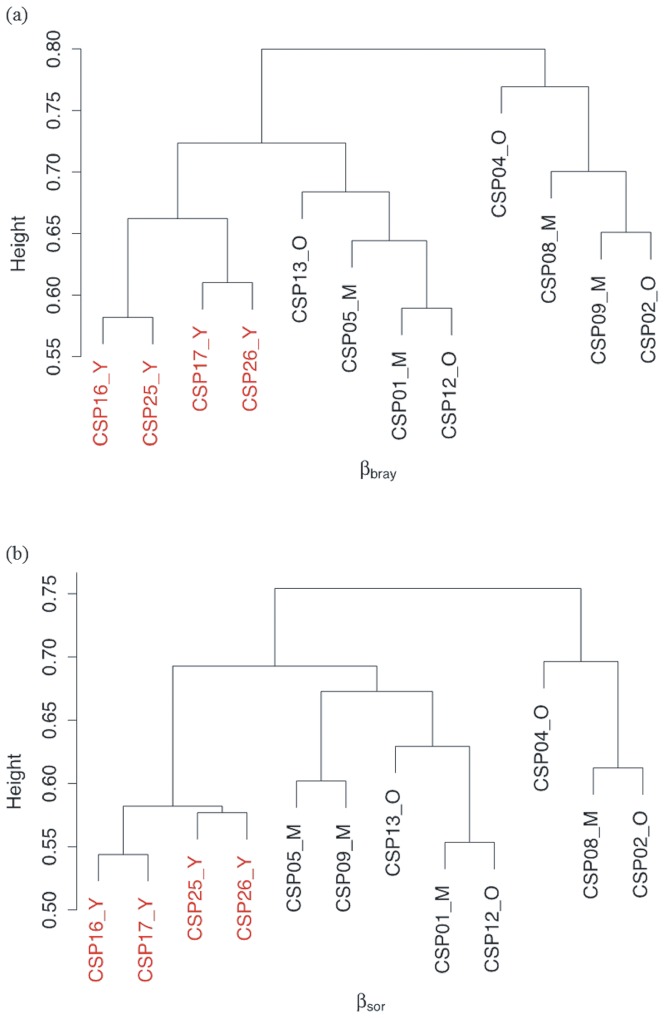
Complete hierarchical clusters based on pair-wise dissimilarity matrices derived from (a) abundance based Bray-Curtis dissimilarity matrix (β_bray_) and (b) incidence based Sorenson dissimilarity matrix accounting for beta diversity (β_sor_). Both cluster diagrams showed that the observed significant effect of forest age to the fungal alpha (adonis R^2^ = 0.235, *P*< <0.01) and beta diversity (adonis R^2^ = 0.231, *P*<0.05) differences is mainly attributed to the fungal communities of the young age class forests.

Both abundance and incidence based pair-wise fungal community dissimilarity analysis indicated that fungal alpha diversity (adonis R^2^ = 0.235, *P*< <0.01) and beta diversity (adonis R^2^ = 0.231, *P*<0.05) were significantly correlated with forest age. Hierarchical cluster analysis of these dissimilarity matrices also revealed a clear cluster of the young forest plots (Fig. 1 a, b). Consistently our results showed the presence of age class related fungal community distribution in this forest ecosystem. We found a total of 20, 15 and 27 ascomycetous and 60, 43 and 49 basidiomycetous unique fungal OTUs in the young, medium and old forest age classes, respectively. The ten most abundant and unique ascomyceteous and basidiomycetous fungal OTUs in each forest age class are presented in Table S2.

**Figure 2:**
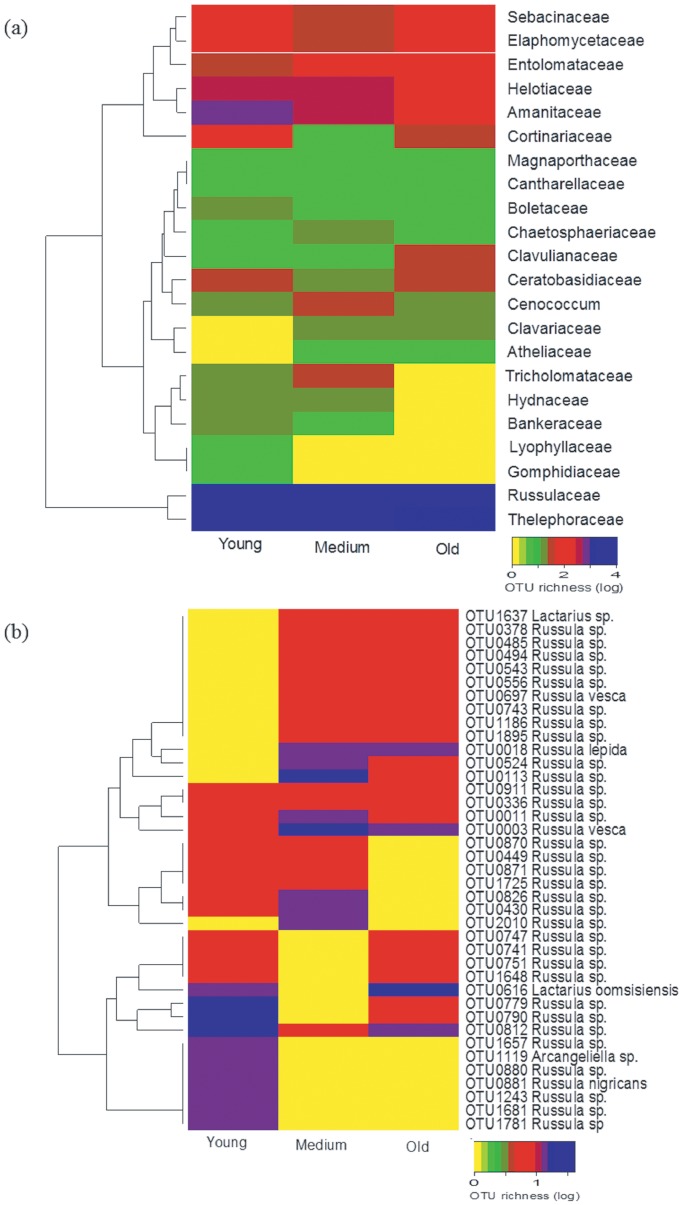
Distribution of observed richness of ECM fungal communities at the family (a) and OTU level of the most abundant ECM fungal family ***Russulaceae*** **(b) across the three forest age classes visualized by heatmap.**

Complete hierarchical cluster analysis based on the relative abundance of the ectomycorrhizal fungal families and visualization using a heatmap also indicated age class related distribution of some ECM families (Fig. 2a). For example, members of the *Lyophyllaceae* and *Gomphidiaceae* were detected only in the young age class forest while *Russulaceae* (142 OTUs) and *Thelephoraceae* (80 OTUs) were the most abundant groups in all the three forest age classes. Similar visualization of ECM fungal OTUs in both families indicated the presence of age class related ECM fungal OTUs (the heatmap for the family Russulaceae is depicted in Fig. 2b). All detected ascomycetous ECM fungi appeared generally across the forest age classes, while some basidiomycetous ECM fungi were found only in the young or shared between the medium and the old forest age classes (Table S3).

### Relationships between Fungal Communities and Environmental Variables

**Figure 3:**
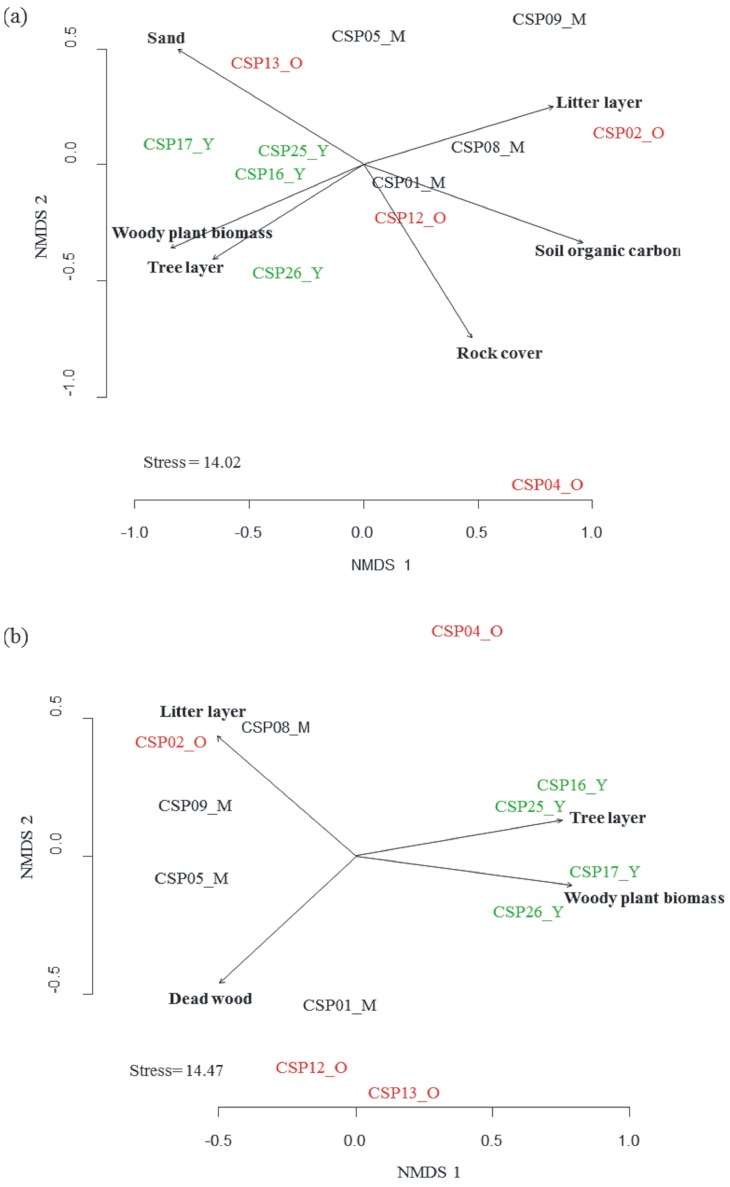
Non-metric multidimensional scaling (NMDS) ordination of the study plots across three forest age classes (Y: Young, M: Medium, O: Old) based on fungal communities of (a) kingdom fungi and (b) ectomycorrhizal fungi. In each diagram, soil and plant characteristics that showed a significant goodness of fit based on post-hoc correlations (*P*≤0.05) are represented as vectors. Stress values represent percentages.

NMDS analysis followed by environmental variable fitting to assess the relationship of individual variables to the ordination plot indicated that the fungal community composition was significantly related to forest age and to plant and soil parameters (Table 2, Fig. 3a). In contrast to the ascomycetous communities the basidiomycetous and ECM fungal community ordinations were influenced by forest age and plant parameters (Table 2, Fig. 3b).

**Table 2:** Goodness of fit statistics or squared coefficients of environmental variables fitted to the Nonmetric Multi-dimensional Scaling (NMDS) ordination space of fungal, Ascomycota, Basidiomycota, and ECM fungal communities.

Environmental variables	Fungi	Ascomycota	Basidiomycota	ECM fungi
Forest age	**0.378** *	0.043	**0.626** **	**0.427** *
Elevation (m)	0.188	0.042	**0.749** **	0.250
Herb layer cover (%)	0.052	0.004	0.266	0.238
Tree layer cover (%)	**0.487** *	0.063	**0.745** **	**0.651** **
Deadwood cover (%)	0.123	0.006	**0.686** **	**0.509** *
Bare soil cover (%)	0.083	0.244	0.242	0.087
Rock cover (%)	**0.637** *	**0.621** *	0.133	0.337
Litter layer (thickness, cm)	**0.613** *	0.312	0.288	**0.494** *
Herb species richness	0.209	0.127	0.484	0.071
Tree species richness	0.418	**0.693** **	0.189	0.307
Herbaceous biomass (g)	0.025	0.137	0.374	0.368
Woody plant biomass (<1 m) (g)	**0.685** **	0.089	**0.630** *	**0.708** **
Litter biomass (dry weight, g)	0.159	0.055	0.103	0.094
Sand (%)	**0.741** **	**0.730** **	0.091	0.386
Clay (%)	0.154	**0.566** *	0.171	0.405
Soil organic carbon (%)	**0.841** ***	**0.638** **	0.184	0.506
C/N	0.382	0.429	0.035	0.031
pH_KCl_	0.342	0.277	0.179	0.460

* *P*<0.05,

** *P*<0.01,

*** *P*<0.001, Fungi = Kingdom Fungi.

Significant correlations (Bonferroni corrected *P*<0.05) are presented in bold.

The dbRDA based model selection, however, indicated SOC and elevation to be the most important factors shaping the fungal community composition (F = 1.63, *P<*0.01) (Table 3). Marginal tests also showed that SOC and elevation were significantly related to the fungal community composition (SOC - F = 1.84, *P<*0.01; elevation - F = 1.42, *P<*0.05). We also found a significant interaction of forest age with SOC and elevation in the fungal (adonis F = 1.37, *P<*0.05) and ascomycetous (adonis F = 2.01, *P = *0.002) communities (Table 3). These results indicated the role of a particular or specific group of environmental drivers that shape the diversity and composition of fungal communities.

**Table 3:** Influence of forest age, elevation of the study site and soil organic carbon (SOC) on the fungal community composition.

Factors	Fungi		Ascomycota		Basidiomycota		ECM fungi	
	F Model	R^2^	F Model	R^2^	F Model	R^2^	F Model	R^2^
Forest age	1.843	**0.135** **	1.424	0.100	2.015	**0.146** **	1.924	**0.136** **
SOC	1.633	**0.120** **	1.883	**0.131** **	1.515	**0.110** *	1.434	**0.102** *
Elevation	1.294	0.095	1.305	0.091	1.407	**0.102** *	1.402	**0.099** *
Forest age:SOC	1.146	0.084	1.271	0.089	1.132	0.082	1.246	0.089
Forest age:Elevation	1.130	0.083	1.208	0.084	1.224	0.089	1.391	**0.098** *
SOC:Elevation	1.182	0.087	1.196	0.084	1.270	0.092	1.448	**0.103** *
Forest age:SOC:Elevation	1.372	**0.100** *	2.013	**0.140** **	1.169	0.085	1.205	0.086

Significant values (*P*<0.05) of the permutational multivariate analysis of variance results are presented in bold.

* P<0.05,

** P<0.01,

*** P<0.001, Fungi = Kingdom Fungi.

### Relationship between Fungal and Tree Communities

The correspondence of the fungal and tree community as revealed by the Procrustes correlation analysis showed a significant relationship across the study sites, at the level of the fungal kingdom (Procrustes correlation coefficient = 0.664, *P*<0.01). Consistently we found a significant correlation of the plant community to the Basidiomycotan and ECM fungal communities. Similar analysis based on ECM and non-ECM tree communities demonstrated a significant agreement with the fungal community. Contrary to our expectations the concordance between ECM fungi and ECM tree communities was not significant (Table 4, Fig. S4). ANOVA based tests to assess the influence of soil characteristics (Table 1) on the relationships between fungal and tree community assemblages showed no significant effect of the soil parameters on the observed concordance between tree and fungal communities (Bonferroni corrected *P*>0.05).

**Table 4:** Correspondence of fungal communities with all plant, ECM plant and non-ECM plant species communities based on Procrustes correlation analysis with Bonferroni corrected *P* values.

Fungal taxonomic level	Plant community	ECM plant community	Non-ECM plant community
Fungal community	*r = *0.664, *P*<0.01	*r = *0.692, *P*<0.01	*r = *0.651, *P*<0.05
Ascomycetous community	*ns*	*ns*	*ns*
Basidiomycetous community	*r = *0.651, *P*<0.05	*ns*	*r = *0.639, *P*<0.05
ECM fungal community	*r = *0.689, *P*<0.05	*ns*	*r = *0.637, *P*<0.05

ns: not significant; r: Procrustes correlation coefficients.

## Discussion

### Fungal Diversity and Taxonomic Assignment

In this study we found evidence for a highly diverse soil fungal community in sub-tropical forests of China. Despite our stringent sequence quality filtering and normalization of sequence reads per sample we found 1027 fungal OTUs (including 457 singletons). The number of fungal OTUs we found in this forest ecosystem, without sequence read normalization as presented in many of the published works, is relatively higher than previously reported fungal diversity records of 1077 fungal OTUs from *Quercus* spp. ectomycorrhizas [60], 47 arbuscular mycorrhizal fungal OTUs from root samples of 10 plant species [61], and up to 1000 fungal OTUs per 30,000 reads from European spruce and beech forest soil [35].

The fungal communities were predominantly members of the phylum Basidiomycota, followed by Ascomycota and Zygomycota. This observation is in accordance with results from European forests [35]. In contrast, in Australian subtropical forests Curlevski et al. [62] showed a dominance of Ascomycota over Basidiomycota and Zygomycota, while He et al. [63] reported Zygomycota as the dominant phylum. Such diverse results may indicate lack of a general global pattern in distribution and abundance of the main fungal phyla in forest soils. Although this study has a limitation due to the relatively low number of final sequences per sample, the observed differences with other studies could also be attributed to differences in DNA extraction protocols, differential amplification of different PCR primers and PCR conditions used in the different studies. This suggests a need for larger scale investigations with standardized approaches [64].

In the investigated Chinese subtropical forest ecosystem dominated by non-ECM trees, which accounts for 78.4% of the plant species, we found 75% of the basidiomycetous and 10% of the ascomycetous OTUs to be putative ECM fungi. The result corroborates the observed prevalence of basidiomycetous ECM fungi based on ECM root analysis in a subtropical forest in Dujiangyan [16] and supports the observation that ECM fungi tend to have higher diversity in mixed forests with a higher proportion of non-ECM tree species as compared to forests composed of exclusively or dominantly ECM trees (e.g. [65]). Here, we observed that *Russulaceae* and *Thelephoraceae* were the most abundant ECM fungal families. This observation has also been documented in tropical [7], [8], [11], subtropical [16], [66], [67], Mediterranean [68], and temperate [16], [30] forest ecosystems, suggesting a global pattern of distribution for these two ECM fungal families.

### Relationships between Fungal Communities and Forest Age Classes

Consistent with previous studies we found a strong influence of forest age on the fungal community composition [16], [18], [69]. Our results showed significant differences in the fungal community of the kingdom fungi, phylum Basidiomycota and ECM fungi between the three age classes. The observed clear cluster of the young forest plots (Fig. 1) is mainly related to the decline in the mean Shannon and Simpson diversity indices of the fungal and ECM communities from young to old age class forests. This indicates that some species might become dominant in the fungal community with time. This observation is consistent with that of Keizer and Arnolds [70] and Wallander et al. [18], who suggested that the ECM fungal diversity increases fairly rapidly in the first 30–40 years of forest development and gradually decreases to an intermediate or rather constant level. However, confirmation of this pattern of change would require a longer successional sequence [17], [71], [72].

Detailed analysis of the ECM fungal communities at the family and genus levels also revealed distinct fungal communities in the three age classes. The observed pattern of ECM fungal distribution indicates the importance of forest age in subtropical forest ecosystems and suggests succession of fungal communities during forest development. This pattern forms a strong contrast to the plant communities, where among the 148 tree species found in our study forest, only one shrub (*Photinia glabra*) and two woody species (*Glochidion puberum, Platycarya strobilacea*) were significantly concentrated in the oldest and youngest forests respectively [32].

### Relationships between Fungal Communities and Environmental Variables

We found a strong relationship between individual environmental variables such as tree layer cover, woody plant biomass, litter layer, dead wood cover, sand and SOC with the fungal community composition. These observations corroborate with previously reported positive correlations between the index of plant primary production with fungal diversity and community composition [73]. In accordance with the known role of soil fungi in decomposition of leaf litter and dead wood in forest ecosystems [22], [74], [75], we also found a significant contribution of the litter and dead wood cover on the basidiomycetous and ECM fungal community composition.

Analysis of the role of these environmental variables on the fungal community composition accounting the effects of other parameters indicated that forest age, elevation, and SOC are the most important variables. This result is in line with our observation of the strong correlation of forest age with tree layer cover, woody plant biomass, and dead wood cover, while SOC is correlated with sand and litter layer (see Table S3). Our finding of elevation as one of the important variables shaping fungal community composition is in agreement with Bahram et al. [20]. Considering the strong correlation between forest age, herb layer cover, and herb species richness with the elevation of the study plots and the suggestion of Bahram et al. [20] to take elevation as a proxy for environmental variables such as precipitation and temperature, our result indicates, the need for further large-scale study considering more climatic variables.

### Relationship between Fungal and Tree Communities

In accordance with our hypothesis, the Procrustes correlation analyses revealed a remarkable congruence between tree and fungal communities, except for the ECM tree and ECM fungal communities. The absence of concordance between ECM fungal and ECM tree communities in this study contradict the reports from boreal forests where close linkages between these groups were documented [25], [76]. This observed lack of correlation could be attributed to our sampling strategy. First although we sampled 9 subsamples per plot, bulking of the samples would have homogenized the fungal diversity and reduced the plant species effect on the fungal community. Second since ECM fungal distribution shows relationship with soil horizons [77], our sampling depth might have restricted the number of plant species specific ECM fungal species recovered in this study. Nevertheless, our results strongly support previous observations in tropical [8] and subtropical [14] forests, and indicated that the spatial pattern of plant communities, proportion of ECM and non-ECM plants as drivers of the fungal community composition.

### Conclusion

This study provides evidence of high soil fungal diversity in this subtropical forest ecosystem. In general our findings suggested the existence of plant and soil related effects on soil fungal community composition. Forest age, elevation of the study plots and SOC were found to be the most important factors influencing the fungal community composition. The observed significant correspondence between tree and fungal communities and the relative contribution of the ECM and non-ECM tree species communities on the fungal community composition suggests the existence of unidentified specific links of resources provision and utilization between plants and rhizosphere fungal communities in this subtropical forest. Elucidating these links will require experimental testing in manipulated systems that vary in plant species diversity, which is the main focus in the ongoing BEF China project.

## Supporting Information

Figure S1 **Distribution of the 12 Comparative Study Plots (CSPs) in the Gutianshan National Nature Reserve (NNR).** CSPs are represented by open circles and labeled according to their age class.
(TIFF)

Figure S2 **Spearman’s rank correlation of the environmental variables.**
(TIFF)

Figure S3 **Relative abundance based distribution of the ten most abundant ECM fungal families (a) and genera (b) across the three forest age classes.**
(TIF)

Figure S4 **Procrustean superimposition plots of plant community ordinations with (a) fungal community, (b) Ascomycotan fungal community, (c) Basidiomycotan fungal community, and (d) ECM fungal community ordination plots.**
(TIFF)

Table S1 **Study plot names, barcodes and sequence reads recovered per sample at different steps of the data analysis.** Trimmed dataset: after sequence quality filtering, barcode and primer and trimming; fungal dataset: Number of sequence reads after non fungal and chimeric sequence removal; Normalized dataset: Sequence reads are normalized per sample; Forward primer: CTTGGTCATTTAGAGGAAGTAA.
(DOCX)

Table S2 **NCBI blastn based taxonomic assignments of the ten most abundant ascomycetous and basidiomycetous fungal OTUs found exclusively in each of the forest age classes.**
(DOCX)

Table S3 **Putative ectomycorrhizal fungal community distribution among the three forest age classes.** Numbers refer to the number of ECM fungal OTUs found from the respective ECM fungal family and forest age class.
(DOCX)

Table S4 **Relationships among the environmental variables based on Pearson correlation analysis.** Significant correlations (*P*<0.05) are in bold.
(DOCX)
