# Supplementary figures and images for: Forest Age and Plant Species Composition Determine the Soil Fungal Community Composition in a Chinese Subtropical Forest
Source: PLoS One. 2013 Jun 27;8(6):e66829. doi: 10.1371/journal.pone.0066829 (PMC3694989; doi:10.1371/journal.pone.0066829)

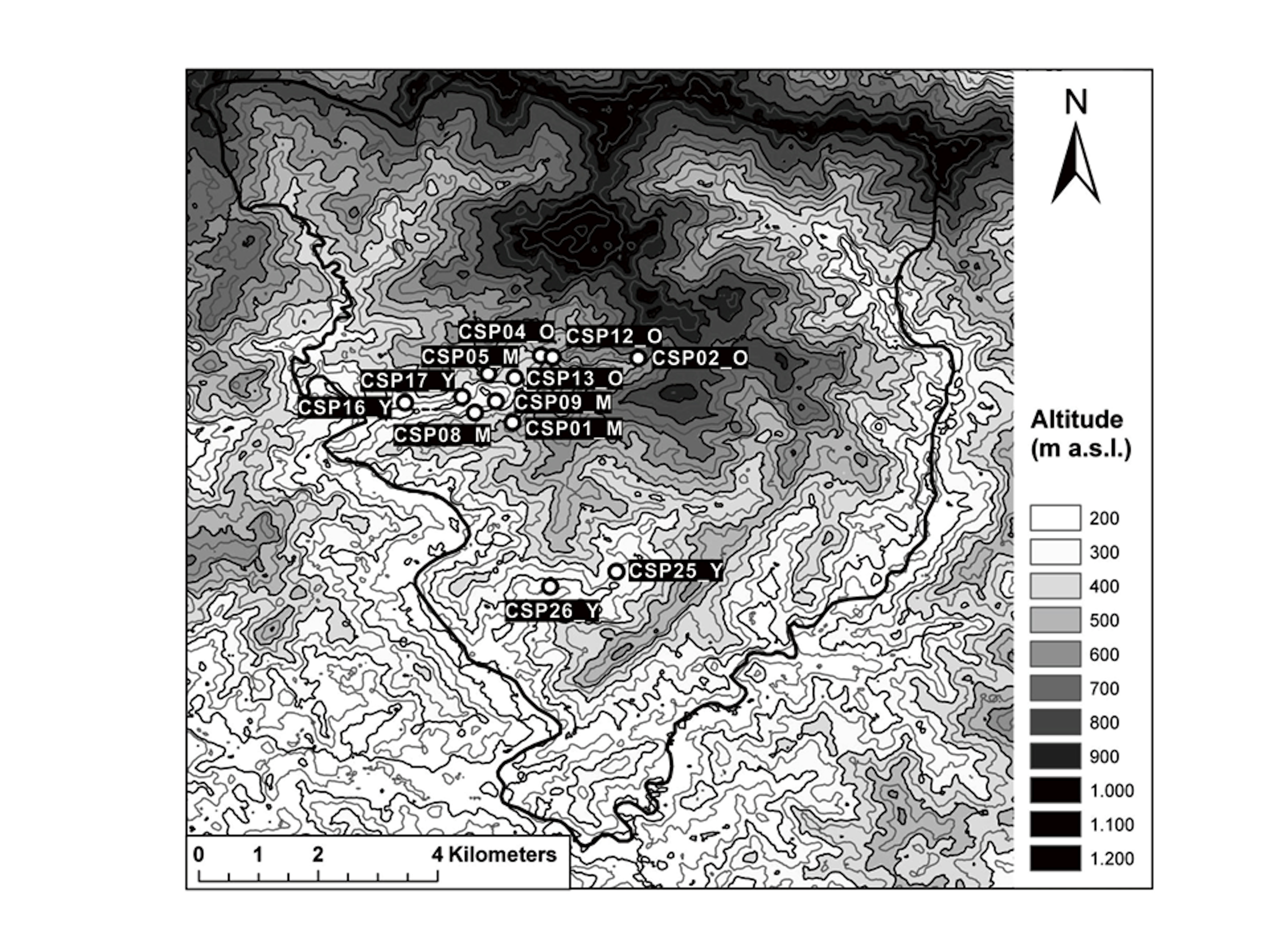

Supplement: Figure S1 — Distribution of the 12 Comparative Study Plots (CSPs) in the Gutianshan National Nature Reserve (NNR). CSPs are represented by open circles and labeled according to their age class. (TIFF) [file pone.0066829.s001.tiff]

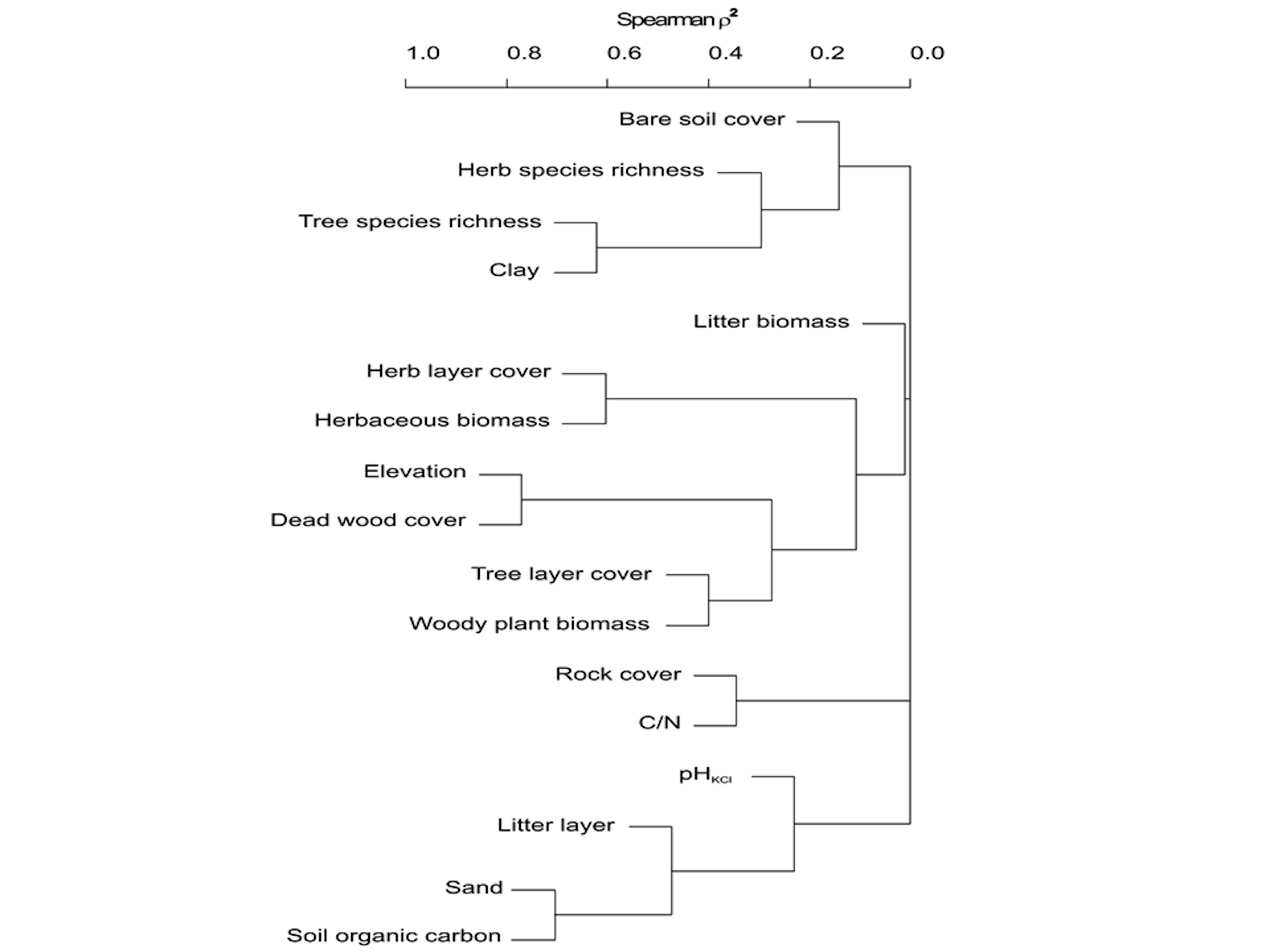

Supplement: Figure S2 — Spearman’s rank correlation of the environmental variables. (TIFF) [file pone.0066829.s002.tiff]

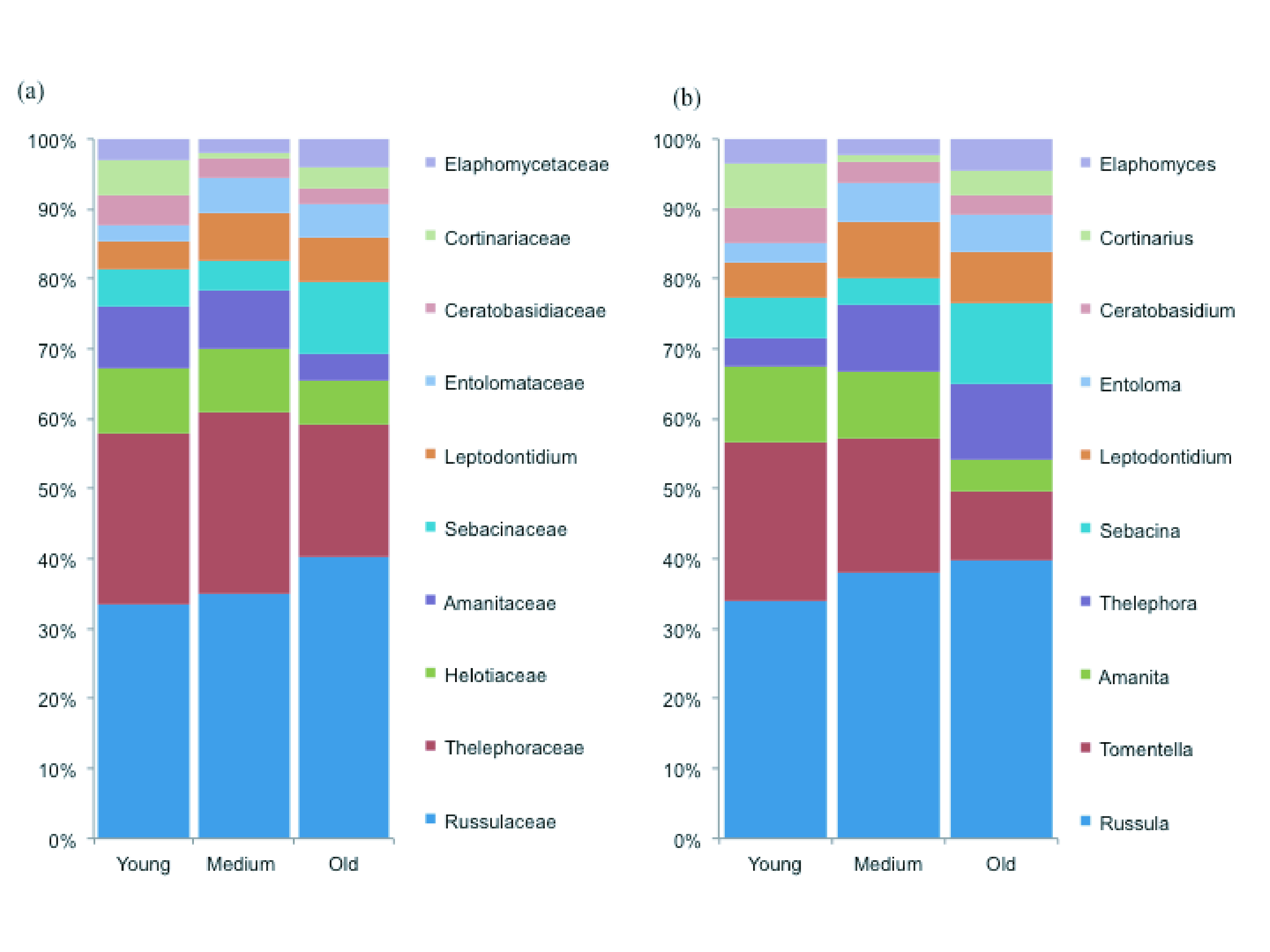

Supplement: Figure S3 — Relative abundance based distribution of the ten most abundant ECM fungal families (a) and genera (b) across the three forest age classes. (TIF) [file pone.0066829.s003.tiff]

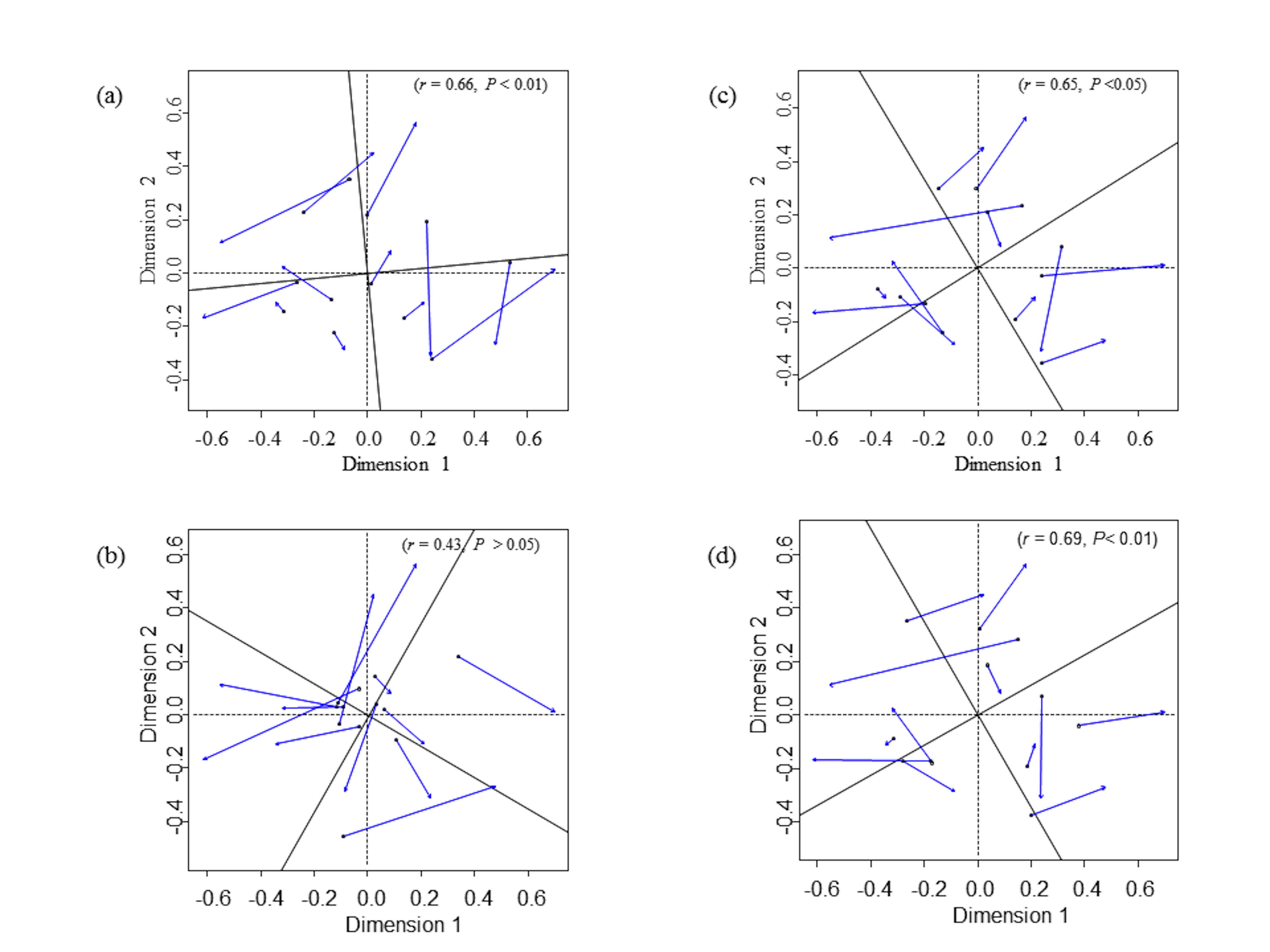

Supplement: Figure S4 — Procrustean superimposition plots of plant community ordinations with (a) fungal community, (b) Ascomycotan fungal community, (c) Basidiomycotan fungal community, and (d) ECM fungal community ordination plots. (TIFF) [file pone.0066829.s004.tiff]
