# Supplementary material for: Forest Age and Plant Species Composition Determine the Soil Fungal Community Composition in a Chinese Subtropical Forest
Source: PLoS One. 2013 Jun 27;8(6):e66829. doi: 10.1371/journal.pone.0066829 (PMC3694989; doi:10.1371/journal.pone.0066829)
Supplement: Table S1 — Study plot names, barcodes and sequence reads recovered per sample at different steps of the data analysis. Trimmed dataset: after sequence quality filtering, barcode and primer and trimming; fungal dataset: Number of sequence reads after non fungal and chimeric sequence removal; Normalized dataset: Sequence reads are normalized per sample; Forward primer: CTTGGTCATTTAGAGGAAGTAA. (DOCX) [file pone.0066829.s005.docx]

**Table S1** Study plot names, barcodes and sequence reads recovered per sample at different steps of the data analysis. Trimmed dataset: after sequence quality filtering, barcode and primer and trimming; fungal dataset: Number of sequence reads after non fungal and chimeric sequence removal; Normalized dataset: Sequence reads are normalized per sample; Forward primer: CTTGGTCATTTAGAGGAAGTAA

|  |  |  | **Sequence reads distribution per sample** | | | | |
| --- | --- | --- | --- | --- | --- | --- | --- |
| **Study plot** | **Forest age** | **Barcodes** | | **Trimmed dataset** | **Fungal dataset** | **Normalized dataset** |  |
| CSP16 | young | TAGTATCAGC | | 1436 | 1185 | 872 |  |
| CSP17 | young | TCTCTATGCG | | 1222 | 1063 | 872 |  |
| CSP25 | young | TGATACGTCT | | 1251 | 991 | 872 |  |
| CSP26 | young | TACTGAGCTA | | 1120 | 954 | 872 |  |
| CSP02 | Old | CATAGTAGTG | | 1605 | 1455 | 872 |  |
| CSP04 | Old | CGAGAGATAC | | 1016 | 977 | 872 |  |
| CSP12 | Old | ATACGACGTA | | 1056 | 958 | 872 |  |
| CSP13 | Old | TCACGTACTA | | 1126 | 1025 | 872 |  |
| CSP01 | medium | CGTCTAGTAC | | 1166 | 1026 | 872 |  |
| CSP05 | medium | TCTACGTAGC | | 1115 | 919 | 872 |  |
| CSP08 | medium | TGTACTACTC | | 1074 | 912 | 872 |  |
| CSP09 | medium | ACGACTACAG | | 949 | 872 | 872 |  |
