# Supplementary material for: Forest Age and Plant Species Composition Determine the Soil Fungal Community Composition in a Chinese Subtropical Forest
Source: PLoS One. 2013 Jun 27;8(6):e66829. doi: 10.1371/journal.pone.0066829 (PMC3694989; doi:10.1371/journal.pone.0066829)
Supplement: Table S2 — NCBI blastn based taxonomic assignments of the ten most abundant ascomycetous and basidiomycetous fungal OTUs found exclusively in each of the forest age classes. (DOCX) [file pone.0066829.s006.docx]

**Table S2** NCBI blastn based taxonomic assignments of the ten most abundant ascomycetous and basidiomycetous fungal OTUs found exclusively in each of the forest age classes
