# Supplementary material for: Forest Age and Plant Species Composition Determine the Soil Fungal Community Composition in a Chinese Subtropical Forest
Source: PLoS One. 2013 Jun 27;8(6):e66829. doi: 10.1371/journal.pone.0066829 (PMC3694989; doi:10.1371/journal.pone.0066829)
Supplement: Table S3 — Putative ectomycorrhizal fungal community distribution among the three forest age classes. Numbers refer to the number of ECM fungal OTUs found from the respective ECM fungal family and forest age class. (DOCX) [file pone.0066829.s007.docx]

**Table S3** Putative ectomycorrhizal fungal community distribution among the three forest age classes. Numbers refer to the number of ECM fungal OTUs found from the respective ECM fungal family and forest age class
