# Supplementary material for: Forest Age and Plant Species Composition Determine the Soil Fungal Community Composition in a Chinese Subtropical Forest
Source: PLoS One. 2013 Jun 27;8(6):e66829. doi: 10.1371/journal.pone.0066829 (PMC3694989; doi:10.1371/journal.pone.0066829)
Supplement: Table S4 — Relationships among the environmental variables based on Pearson correlation analysis. Significant correlations (P<0.05) are in bold. (DOCX) [file pone.0066829.s008.docx]

**Table S4** Relationships among the environmental variables based on Pearson correlation analysis. Significant correlations (*P* < 0.05) are in bold.

|  | Age | Elevation | Clay | Sand | corg | C:N ratio | pHKCl | Herb layer cover | Herb species richness | Herbaceous biomass | Tree layer cover | Tree species richness | Woody plant biomass | Dead wood cover | Litter layer | Litter biomass | Bare soil cover |
| --- | --- | --- | --- | --- | --- | --- | --- | --- | --- | --- | --- | --- | --- | --- | --- | --- | --- |
| Elevation | **0.705** |  |  |  |  |  |  |  |  |  |  |  |  |  |  |  |  |
| Clay | 0.015 | -0.362 |  |  |  |  |  |  |  |  |  |  |  |  |  |  |  |
| Sand | **-0.611** | -0.359 | **-0.654** |  |  |  |  |  |  |  |  |  |  |  |  |  |  |
| corg | 0.535 | 0.413 | 0.466 | **-0.866** |  |  |  |  |  |  |  |  |  |  |  |  |  |
| C:N ratio | -0.364 | -0.026 | -0.562 | **0.639** | -0.444 |  |  |  |  |  |  |  |  |  |  |  |  |
| pHKCl | -0.038 | -0.009 | -0.528 | 0.524 | **-0.586** | -0.074 |  |  |  |  |  |  |  |  |  |  |  |
| Herb layer cover | -0.427 | **-0.588** | 0.155 | 0.162 | -0.044 | 0.475 | -0.47 |  |  |  |  |  |  |  |  |  |  |
| Herb species richness | -0.271 | **-0.684** | 0.522 | 0.046 | -0.066 | -0.22 | -0.202 | 0.368 |  |  |  |  |  |  |  |  |  |
| Herbaceous biomass | -0.512 | -0.546 | 0.389 | -0.033 | 0.187 | 0.26 | **-0.685** | **0.83** | 0.38 |  |  |  |  |  |  |  |  |
| Tree layer cover | **-0.754** | -0.56 | 0.168 | 0.288 | -0.322 | 0.001 | 0.187 | 0.278 | 0.204 | 0.292 |  |  |  |  |  |  |  |
| Tree species richness | 0.173 | -0.256 | **0.762** | **-0.585** | **0.673** | -0.574 | -0.449 | 0.164 | **0.596** | 0.419 | -0.039 |  |  |  |  |  |  |
| Woody plant biomass | **-0.631** | -0.432 | 0.018 | 0.492 | **-0.64** | 0.245 | 0.222 | 0.201 | 0.04 | 0.203 | **0.638** | -0.324 |  |  |  |  |  |
| Dead wood cover | **0.712** | **0.877** | -0.429 | -0.213 | 0.342 | -0.141 | 0.256 | **-0.726** | -0.564 | **-0.674** | **-0.594** | -0.127 | -0.548 |  |  |  |  |
| Litter layer | 0.429 | 0.196 | 0.291 | -0.499 | **0.739** | 0.07 | **-0.617** | 0.379 | 0.133 | 0.438 | -0.427 | **0.618** | -0.524 | 0.167 |  |  |  |
| Litter biomass | -0.099 | 0.052 | 0.255 | 0.01 | -0.066 | -0.078 | 0.031 | -0.288 | -0.146 | -0.014 | 0.052 | 0.062 | 0.566 | 0.027 | -0.116 |  |  |
| Bare soil cover | 0.04 | -0.176 | 0.46 | -0.203 | 0.195 | **-0.593** | 0.048 | -0.456 | 0.471 | -0.162 | -0.007 | 0.574 | -0.271 | 0.141 | -0.027 | 0.132 |  |
| Rock cover | 0.432 | 0.244 | 0.473 | **-0.741** | 0.493 | **-0.695** | -0.146 | -0.31 | -0.086 | -0.165 | -0.22 | 0.295 | -0.215 | 0.078 | 0.049 | 0.141 | 0.034 |
